# Workgroup Report: Biomonitoring Study Design, Interpretation, and Communication—Lessons Learned and Path Forward

**DOI:** 10.1289/ehp.8197

**Published:** 2005-07-06

**Authors:** Michael N. Bates, Joshua W. Hamilton, Judy S. LaKind, Patricia Langenberg, Michael O’Malley, Wayne Snodgrass

**Affiliations:** 1 School of Public Health, Division of Environmental Health Sciences, University of California, Berkeley, California, USA; 2 Center for Environmental Health Sciences and Department of Pharmacology and Toxicology, Dartmouth Medical School, Hanover, New Hampshire, USA; 3 LaKind Associates, LLC, Catonsville, Maryland, USA; 4 Milton S. Hershey Medical Center, Penn State College of Medicine, Hershey, Pennsylvania, USA; 5 Department of Epidemiology and Preventive Medicine, School of Medicine, University of Maryland, Baltimore, Maryland, USA; 6 Employee Health Services, University of California, Davis, California, USA; 7 University of Texas Medical Branch, Galveston, Texas, USA

**Keywords:** biomonitoring, communication, design, human health, interpretation, specimen archiving

## Abstract

Human biomonitoring investigations have provided data on a wide array of chemicals in blood and urine and in other tissues and fluids such as hair and human milk. These data have prompted questions such as *a*) What is the relationship between levels of environmental chemicals in humans and external exposures? *b*) What is the baseline or “background” level against which individual levels should be compared? and *c*) How can internal levels be used to draw conclusions about individual and/or population health? An interdisciplinary panel was convened for a 1-day workshop in November 2004 with the charge of focusing on three specific aspects of biomonitoring: characteristics of scientifically robust biomonitoring studies, interpretation of human biomonitoring data for potential risks to human health, and communication of results, uncertainties, and limitations of biomonitoring studies. In this report we describe the recommendations of the panel.

Environmental health sciences focus on the relationship between exposures to environmental chemicals of concern and their relationship to health outcomes. The traditional method for assessing human exposures to environmental chemicals is to estimate, by empirical or modeling methods, the concentrations of chemicals of potential concern in environmental media (air, water, soil, food), and then combine this information with estimates of human exposure (e.g., estimates of daily consumption of tap water) to determine a dose. However, as analytic techniques have evolved, there has been an increasing focus on development and use of human biomonitoring (i.e., measurements of levels of environmental chemicals in human fluids such as blood, urine, or milk, and in tissues such as hair, nails, and fat) for evaluating exposure. These data have often supplemented or even supplanted estimates of exposure based on environmental measures.

Biomonitoring has been used for several decades for certain chemicals, such as for lead in blood and cotinine in urine. More recently, biomonitoring has provided data on levels of a much wider array of chemicals in various human fluids and tissues. In the United States, a systematic program of biomonitoring by the Centers for Disease Control and Prevention (CDC) has resulted in its National Report on Human Exposure to Environmental Chemicals (CDC 2003). This and other biomonitoring-based research have produced a substantial database on levels of environmental chemicals in humans. However, important questions remain: What is the relationship between these internal levels and external exposures? What is the baseline or “background” level against which individual levels should be compared? And how can internal levels be used to draw conclusions about individual and/or population health?

Uncertainties related to the relationships of exposure with internal dose and of internal dose with potential for adverse health effects have been described by the CDC (CDC 2003) and others (LaKind et al. 2005; Sexton et al. 2003; Stokstad 2004). These uncertainties were also highlighted at a recent workshop on environmental chemicals in human milk (LaKind 2005), during which a multidisciplinary group addressed questions regarding interpretation of human milk biomonitoring data for both the health of the mother and the breastfeeding infant. Recognizing that individuals from an array of disciplines have been grappling with various aspects of biomonitoring and that these disparate disciplines bring different perspectives to the table, the Research Foundation for Health and Environmental Effects (RFHEE; a co-sponsor of the human milk biomonitoring workshop) convened an interdisciplinary panel for a 1-day workshop on 13 November 2004 with the charge of focusing on three specific aspects of biomonitoring: characteristics of scientifically robust biomonitoring studies, interpretation of human biomonitoring data for potential risks to human health, and communication of results, uncertainties, and limitations of biomonitoring studies. The RFHEE sought panel members with expertise in medicine, toxicology, epidemiology, biostatistics, and risk assessment (the authors of this paper formed the workshop panel). During the workshop, the panel drew from the fields of medicine and occupational health, which have a long history of research on biomonitoring [National Institute for Occupational Safety and Health (NIOSH) 2004], as well as on the interpretation of the implications of biomonitoring results for individuals. In this report, we describe the recommendations reached during the workshop regarding biomonitoring study design, interpretation, and communication.

## Recommendations of the Workshop Panel

### Biomonitoring Study Design

The design of any scientific study depends on its goals and hypotheses. However, fundamental features that make for scientifically robust and credible studies exist. The panel focused on key research needs and recommendations for ensuring that the goals, hypotheses, and study design parameters are realistic, transparent, and scientifically robust.

#### Investigators need to gain not only approval but also acceptance for human studies.

Biomonitoring studies involving human subjects “must be conducted in a way that protects subjects’ rights and well-being,” and must have the oversight of an institutional review board (IRB), as described by the Common Rule, the International Conference on Harmonisation Good Clinical Practice guidelines, and the Declaration of Helsinki (LaKind et al. 2005). Nevertheless, biomonitoring studies may prove controversial even when approved by one or more IRBs. A recent example is a U.S. Environmental Protection Agency (EPA) study to monitor children’s exposure to pesticides [the Children’s Environmental Exposure Research Study (CHEERS); CHEERS 2005]. Although the U.S. EPA study involved monitoring rather than experimental administration of pesticides and had been approved by four separate IRBs, criticism of the CHEERS study raised by nongovernmental organizations focused on payments of up to $1,000 to subject families. This was criticized as undue inducement; criticism was also directed at partial industry funding of the study (Environmental Working Group 2004; Pesticide Action Network Updates Service 2004). In response, the U.S. EPA has cancelled the study (U.S. EPA 2005). This example highlights the need for thorough institutional review of human biomonitoring studies and the need for stakeholder acceptance, even after IRB approval is complete. Recommendations regarding ethical restrictions for biomonitoring studies have been developed (Oleskey et al. 2004). Although these were developed for pesticides, many of the recommendations have broader applicability. Researchers should also anticipate controversy from organizations and individuals who may not accept the authority of the IRBs to judge the ethical questions raised by proposed research plans.

#### Because human tissue banking is a critical component of biomonitoring studies, protocols for ensuring sample integrity and access are needed.

Banking of human tissue/fluid samples is important for future hypothesis testing, but it is complicated by cost, space, ethical consent (especially for genomic studies), and Health Insurance Portability and Accountability Act regulations (HIPAA 1996). Proper long-term storage conditions for specimens must be determined. The most useful matrices (e.g., blood, urine, hair) should be targeted for storage and should be linked to individual-level data (e.g., questionnaires, health information). In addition, to maximize the usefulness of such a resource, a mechanism would be needed to provide for future access to the specimens by investigators not necessarily involved in the original study.

#### Prioritization of chemicals selected for biomonitoring studies should be health-based but should also take into account factors such as the ability to bioaccumulate, known exposures in susceptible populations, analytic capabilities, and ability to collect and analyze the matrices that are most appropriate for each chemical of concern.

Put simply, chemicals should not be measured in humans merely because the analytic capability exists. Prioritization should be based on criteria that would yield the most useful data for maximizing public health gain.

An example of a model for prioritization of chemicals for biomonitoring is the recently awarded state biomonitoring program planning grant by the CDC to the state of New Hampshire. New Hampshire was awarded this grant for their proposal to develop biomonitoring programs to evaluate exposures to mercury in fish, MTBE (methyl *tert*-butyl ether) and arsenic in drinking water, and phthalates from plastics. The rationale for selecting these particular chemicals included several factors. First, these chemicals were among the top 10 environmental chemicals of greatest public health concern to that state’s population (New Hampshire has high levels of arsenic in drinking water and high levels of mercury in fish, and recognizes the growing incidences of MTBE groundwater contamination and expanding database on phthalate exposures in the general population). Also, there were readily available and validated methods for analysis of these chemicals in the appropriate biologic matrices in the population (arsenic and mercury in nails, MTBE and phthalates in urine or blood), which could be readily obtained, stored, and analyzed. Finally, there were known links to human health effects at exposures of concern (arsenic, mercury), and/or there was growing concern about potential health effects due to widespread or increasing exposure (arsenic, mercury, MTBE, phthalates) and potential for biomagnification in the food chain or accumulation in humans (mercury). Other chemicals of concern lacked one or more of these key attributes: They were not of unique or particular concern to New Hampshire, were not known to bioaccumulate, lacked an appropriate, practical or cost-effective methodology to analyze accurately, required specialized matrices (e.g., fat biopsy) that were impractical to obtain for a population study, or there was little or no toxicologic information to directly implicate them in specific health effects in humans. This selection process serves as an excellent paradigm for chemical selection for future biomonitoring studies.

#### Collaborations among academia, industry, and government should be encouraged to facilitate biomonitoring studies in occupational settings.

A critical component of any occupational epidemiology study is the assessment of exposure. Often industry has carried out its own monitoring, including biomonitoring of the workforce, as part of the routine assessment of employee exposure, with results residing in files or archives. However, investigators from outside the industry typically must rely on more limited data for exposure estimation, such as dates of employment, possibly obtained from union records. Collaborations with industry, built on mutual trust and respect, can often mean the availability of more specific exposure data. These can include biomonitoring results as well as environmental monitoring results and job-related data. For example, in a recent cohort study of chlorpyrifos manufacturing workers at the Dow Chemical Company, serial measurements were made over the study period of the chlorpyrifos urinary metabolite 3,5,6-trichloro-2-pyridinol and the intermediate end point, cholinesterase, allowing the investigators to carefully characterize occupational exposures and examine potential associations with health outcomes (Albers et al. 2004). A potential drawback for extrapolating these types of results (linking exposure to levels in humans) to the general population is that these occupational exposures may not be representative of exposures that would be estimated based on monitoring of air and other media, as workers often use protective clothing or work with largely enclosed processes.

#### The panel recommends that investigators consider the following biomonitoring study design issues:

##### Sampling frame.

The extent to which results can be generalized to a wider population will depend almost entirely on the sampling frame of the study. The best sampling frame is a random selection from a clearly defined population (with a high level of participation agreement), which will make results almost entirely generalizable to that population. The extent to which generalization from the study population to the general population will be restricted depends on whether *a*) the population is self-selected (e.g., volunteers), *b*) there is a high degree of nonparticipation, and *c*) participants are selected based on specific characteristics or features (e.g., pregnant women).

##### Laboratory techniques.

The laboratory performing the analyses should conduct rigorous quality assurance/quality control procedures in accordance with accepted guidelines, including calibration of instruments, running appropriate standards and blanks, performing spiking, blinded repeat and other quality assurance measures, and reporting limits of detection, variation, and other statistical parameters along with the experimental results (Needham and Wang 2002; Needham et al. 2002).

##### Selection of human specimen type.

In selecting the matrix for a biomonitoring study, the researcher must fully understand the utility and quality of that matrix, as well as the biologic significance and complexity. This selection is often a balance between purely scientific issues and the practicality of obtaining the matrix for a population study in a cost-effective way that is least invasive and risky to the study participant. However, matrices such as urine or blood, while easy to obtain or available from previous studies, are often not appropriate for measurement of certain chemicals.

##### Integrity of samples.

Improper collection, transportation, and/or storage of specimens can significantly affect the biomonitoring results (Aitio and Jarvisalo 1985; Griffin 1986; Wax et al. 2000). Biomonitoring studies should follow validated protocols that are appropriate to the matrix and analyte(s) of interest. Many samples require collection into the appropriate storage container, clean techniques for handling and storage, chemical or physical stabilization of the sample, storage under appropriate conditions, and control of the number of times a sample is removed from storage and assayed. Failure to follow these steps can produce either degradation or speciation change of the analyte or contamination of the sample from external sources (Versieck 1985). For example, until the past decade or so, it was not widely appreciated how easily a sample can become externally contaminated with chromium, principally from stainless steel and other metal components, but also from other sources. Simply dissecting and then homogenizing a rat liver in a typical laboratory blender can result in chromium contamination many times the actual biologic level in the sample. As a result, most published studies on chromium levels in human and animal tissues that did not recognize this problem contained largely inaccurate data. Analysis of trace levels of metals and other environmental chemicals often requires full clean techniques including metal-free, acid-washed sample collection and storage procedures and special clean-room analysis protocols. Likewise, collection of blood into a typical vacutainer can highly influence analysis of cadmium, plasticizers, and other compounds that may leach from rubber stoppers and walls of containers. Investigators should standardize and report their sample protocols because this can be critical in interpreting and comparing results across studies.

##### Reporting of nondetects.

The method used to assign a value to analytic results below the limit of detection (LOD; e.g., LOD/2) should be described because the method chosen can have an appreciable effect on the results and their interpretation (Helsel 1990). This is especially important in assessing populations in which biomonitored chemical concentrations are frequently below the detection limit. Ideally, a sensitivity analysis should be carried out to determine the effect that the method of assigning values below the LOD has on results and their interpretation.

### Interpretation of Human Biomonitoring Data

Carefully conducted human biomonitoring studies serve several important functions, including *a*) evaluating time trends for levels of environmental chemicals in humans; *b*) evaluating the efficacy of regulatory action; *c*) assessing regional differences in levels of chemicals in populations; and *d*) establishing baselines and distributions of body burdens for populations. Biomonitoring information lends itself well for such interpretation, particularly for studies that are representative of populations of interest. However, besides using biomonitoring data as a marker of exposure, there is intense interest in using these data as markers of health effect(s) and establishing health-related reference levels for the measured chemicals. Alcohol is an example of a chemical for which exposure can be linked to internal dose and internal dose can be linked to effect. Lead is an example of an environmental chemical for which exposures are involuntary and for which there are sufficient data to draw associations between biomonitoring data and health, both to the individual and to a population. Decades of epidemiologic and toxicologic research on lead effects provide the underpinnings for the interpretation of blood lead level information. Not only has interpretive information been made available to the public for lead in children’s blood, but advice on medical interventions has also been developed. Both reference levels and recommended clinical interventions for other environmental chemicals are likely to evolve over time as new information is obtained; it is important to develop and make available such interpretive documents for the medical profession and the public.

Despite public pressure to provide more immediate interpretations of biomonitoring data in terms of potential for impacts on health, the development of analytic methods has provided the ability to measure extraordinarily low concentrations of a wide array of chemicals for which there are insufficient data on which to base those interpretations. In addition, it is often forgotten that the “measurement of an environmental chemical in a person’s blood or urine does not by itself mean that the chemical causes disease” (CDC 2003, p. 2). The panel discussed several issues related to interpretation of biomonitoring data, described below.

#### To interpret biomonitoring data in terms of health, studies are needed on the relationships between exposure and levels in the body and between levels in the body and health effect.

The first critical step is to develop an understanding of the relationship between exposure (applied dose) and body burden. This relationship can be complex, as in the case of arsenic. Although many toxic metals, such as lead, mercury, and cadmium, accumulate in human tissues such that biomonitoring can reveal the extent of long-term cumulative exposure, arsenic and other metals do not accumulate in this way. Each metal has unique pharmacokinetic and pharmacodynamic properties. For arsenic, the exposure of concern is consumption of inorganic arsenic in contaminated drinking water. Arsenic does not bioaccumulate and is readily excreted by the body with a half-life of days. As a result, total arsenic levels in blood and urine are highly variable. Moreover, because the inorganic forms are of primary toxicologic concern, if blood or urine analyses do not speciate arsenic into its various forms, the resulting data often have only limited toxicologic interpretative value. In contrast, arsenic in nails can be an excellent biomarker of exposure because it principally measures inorganic arsenic exposure, integrates exposure over several weeks, is a highly stable matrix, and is relatively impervious to external contamination or other confounders (Karagas et al. 1996, 2000, 2001a, 2001b; Nichols et al. 1998). Toenail arsenic levels were shown to closely correlate with drinking water arsenic at water levels > 1 ppb (Karagas et al. 2000). More important, toxicologic end points of concern were actually better correlated with toenail arsenic than with drinking water arsenic levels, which indicates that, in this particular situation, this bio-marker provided a more precise internal measure of individual exposure than combining measures of external levels (drinking-water arsenic) with estimates of exposure (how much water consumed per day). Such internal measures will also better integrate potential individual differences in uptake, distribution, metabolism, and excretion. This example highlights the many potential advantages of a good biomarker of exposure but also illustrates the importance of determining the appropriate matrix and methodology and the validation of this marker by comparison to external measures of exposure.

The second important step for a validated biomarker is to relate internal body burden, as assessed by the biomarker, to one or more biologic end points, such as changes in gene or protein expression, alterations in enzyme function, and specific polymorphisms or other genotype information. For example, individual arsenic toenail levels have been shown to correlate with decreases in lymphocyte expression of several DNA repair enzymes (Andrew et al. 2003a, 2003b). Such information not only provides potential biomarkers of exposure and effect but also provides direct support for the hypothesis that arsenic influences cancer risk, at least in part, by suppressing DNA repair and thereby increasing the risk from exposure to other environmental agents of concern such as sunlight (for skin cancer) and cigarette smoke chemicals (for lung and bladder cancer) (Karagas et al. 2001a, 2001b, 2004).

#### To be able to interpret biomonitoring data, laboratory reporting of clinical reference levels must be harmonized.

Even for some long-used markers of exposure, such as acetyl-cholinesterase and butyryl cholinesterase, reference levels have not been determined across laboratories using a consistent method (Wilson et al. 2002). There is widespread clinical confusion regarding the interpretation of reference levels, occurring most commonly when appropriate reference values are not provided by a reporting laboratory.

A recent clinical case involving a 46-year-old gardener with chronic malaise illustrates this point. An attending physician ordered a hair analysis for multiple mineral elements, including mercury and 16 additional “nutrient” elements. The gardener’s only reported exposure was having previously worked in cleaning laboratories at a nearby university where elemental mercury was used in manometers and other instruments. His hair mercury concentration was reported as 4.57 ppm, compared to the diagnostic laboratory’s reference range of 0–0.6 ppm. However, the reliability of such hair mineral analysis has been questioned (Barrett 1985; Seidel et al. 2001; Steindel and Howanitz 2001). Because of the reported abnormality of the hair mercury analysis, a repeat 24-hr urine was collected after a DMPS (sodium salt of 2,3-dimercapto-1-propane sulfonic acid) chelation challenge. The urine mercury level was 3.1 μg over the 24-hr period, or 0.83 μg/L (laboratory reference range was reported as 0–4 μg/24 hr). The measured level was similar to the mean level of urine mercury (0.77 μg/L) reported from the most recent National Health and Examination Nutrition Survey (NHANES) study and well below the 90th percentile level (3.15 μg/L) (CDC 2003). Urine mercury concentrations in humans > 100 μg/L have been associated with minor neurologic signs (Goldman et al. 2001), and urine mercury levels > 300 μg/L usually are associated with overt symptoms (Bates 1998). None of these indications was present in the cited case, but the confusion regarding the appropriate reference range may have provoked the decision to treat with DMPS. This case highlights the need for clear and consistent reference values for biomonitoring data on environmental chemicals.

#### Even if data on both toxicity and levels of environmental chemicals in laboratory animals are available for a given chemical, investigators should proceed with caution when attempting to link such information directly with human biomonitoring data and human health effects.

**Figure 1:**
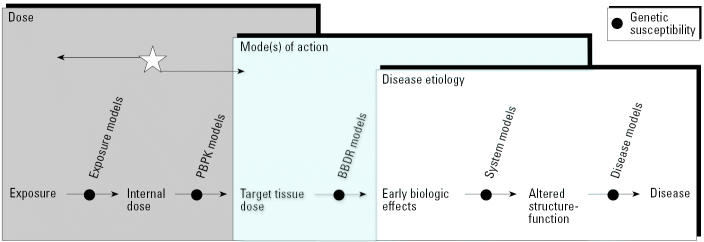
The continuum from exposure to adverse health effect. BBDR, biologically based dose response. With existing toxicologic and epidemiologic databases, we can more readily begin at the internal dose [identified by the star (e.g., biomonitoring data for environmental chemicals in blood)] and move along the arrow to the left, by using models such as PBPK models to obtain information on dose (exposure). At present, for most environmental chemicals, the greater challenge is to begin at the internal dose starting point and move to the right to obtain information about target tissue dose, biologic effects, and disease. (Adapted from Waters and Fostel [2004], with permission from the authors and from the *Nature* Publishing Group.)

Because of species-specific variation in absorption, distribution, metabolism, and elimination, it is difficult to interpret measured levels of environmental chemicals in humans on the basis of levels of chemicals in laboratory animals. However, the goal is to eventually link animal or other laboratory data to human biomonitoring data in a way that harmonizes these data sets for more accurate and robust risk assessments. Currently, the primary approach for linking laboratory animal data to human health effects is via the risk assessment process, which relies on estimates of dose. The no observed adverse effect level (NOAEL) or the lowest observed adverse effect level (LOAEL) is used to establish a “safe” dose for humans. For some chemicals, human biomonitoring information can be used in combination with physiologically-based pharmacokinetic (PBPK) models to estimate dose (the success of this method depends on the properties of the environmental chemical and the availability of parameter information for building the PBPK model). In this way, biomonitoring data can serve as a marker of exposure (Figure 1). However, this is not equivalent to using biomonitoring data as a biomarker of effect. As noted by Bernard and Hermans (1997), biomarkers of early health effects should be stable in the biologic specimen, specific for the target tissue or cell, and sensitive to level of exposure to an insult. In an ideal world, it is desirable to have biomonitoring data serve as both biomarkers of exposure (Figure 1, left arrow) and effect (Figure 1, right arrow). The science is currently insufficiently developed for both of these purposes for most chemicals.

Environmental chemicals are a part of the public health spectrum. Because of the complex nature of disease etiology, scientists will need to obtain and analyze extensive amounts of data to fully understand which environmental chemicals, and at what levels in the body, are linked to adverse health outcomes. A key factor that is often overlooked is the inherent genetic variability of the population, which can profoundly influence disease risk even given the same environmental exposures. The study of gene–environment interactions will require an integration of exposure assessment (such as with biomonitoring) with genetic susceptibility assessment (such as with genetic polymorphism biomarkers). According to Waters and Fostel (2004, p. 943),

Predicting potential human health risks from chemical stressors raises three general challenges: the diverse properties of the thousands of chemicals and other stressors that are present in the environment; the time and dose parameters that define the relationship between exposure to a chemical and disease; and the genetic and experiential diversity of human populations and of organisms used as surrogates to determine the adverse effects of a toxicant.

#### Biomonitoring environmental chemicals is principally a public health tool that is part of the risk assessment framework and, with certain exceptions, is not yet informative as a marker for clinical risk.

Therefore, biomonitoring—especially the types of biomonitoring studies focused on emerging chemicals of concern for which there are limited epidemiologic and toxicologic data—is not generally useful for predicting adverse health effects to the individual. There may be cases, however, where an individual has very high, clinically relevant levels of a given chemical that could be used to assess their individual risk. For example, detection of high arsenic levels in individuals in New Hampshire as part of ongoing epidemiology and biomonitoring studies has been used as the basis for intervention in the form of suggestions for further testing and analysis for those individuals, recommendations to remediate their water, and providing additional resources for information about health effects and remediation options (M. Karagas, personal communication). However, it is important to note and to convey to such individuals that estimates of risk are based on population studies, whereas individual risks will be highly influenced by genetic background, other environmental or occupational exposures, lifestyle factors, and other individually variable factors.

Cardiovascular epidemiology provides a clear example of the complexity of documenting the usefulness and limitations of newly identified markers for clinical risk. The observation of advanced and early-onset atherosclerosis in patients with homocysteinuria prompted evaluation of plasma homocysteine as a risk factor for atherosclerosis in the broader population. An initial case–control study evaluated the level of homocysteine in patients with coronary artery disease compared to normal controls and observed significant differences in mean levels between the two groups (Kang et al. 1986). This finding by Kang et al. has been subsequently confirmed in other retrospective and cross-sectional studies (Braunwald et al. 2001). However, the results from prospective studies have been less consistent (Essebag et al. 2003). Some debate whether modestly elevated plasma homocysteine is a consequence rather than a cause of atherosclerosis (Christen 2000). Randomized trials to test whether lowering homocysteine levels will decrease risks of cardiovascular disease have shown some benefit in some subgroups of patients with preexisting coronary disease. Ongoing research is evaluating the association between homocysteine and stroke and other neurologic outcomes (Huang 2004; Wright et al. 2004).

#### For adequate interpretation of population-based biomonitoring data, improved population-based health data collection is essential.

Most countries have some form of death registration which is usually available to researchers. Some countries or local regions have disease-specific registries, most commonly cancer and birth defect registries. However, to make possible the interpretation of biomonitoring data, other registries are needed for health end points such as neurode-generative and respiratory effects. Newly developed registries should have a rigorous quality assurance program to ensure completeness and accuracy of records.

### Communicating Results of Biomonitoring Studies

Researchers typically use the peer-reviewed scientific literature as their primary method of communication of their study design, results, and interpretation. This method is excellent for communicating with other researchers, especially those in similar disciplines. However, individuals outside of those fields will not necessarily seek out the same journals for information. On occasion, the media will become aware of an emerging scientific issue or publication, and a condensed and simplified version of the information will appear in a form available to the general public.

The scientific community must recognize the public interest in human biomonitoring studies and recognize that this interest has an effect on regulators, federal and state legislators, advocacy organizations, industry, and clinicians (who frequently find themselves on the front lines attempting to address concerns about the meaning of those studies for their patients’ health). Thus, researchers should consider venues in addition to the peer-reviewed literature so that their studies are properly communicated to relevant audiences. One risk associated with failing to do so is the potential misinterpretation of study conclusions by the media and the public. Misinterpretation is even more likely when communicating to a public that needs to be better informed about disease etiology and its multifactorial nature. General information on best practices for risk communication is available in the published literature and from government agencies [Agency for Toxic Substances and Disease Registry (ATSDR) 2001; Fischhoff 1995; National Research Council 1989; Sandman 1990]. Some key points from the workshop follow.

#### Investigators should be able to communicate epidemiologic concepts such as the difference between absolute and relative risk.

For example, in the pediatric condition known as Reye’s syndrome, there is an approximately 4-fold increased relative risk associated with the use of salicylates. But even following salicylate use, Reye’s syndrome remains a rare condition in absolute risk terms, with only 25 cases occurring in the United Sates in 1989 (a population incidence of 1.3 per million children under 5 years of age; Forsyth et al. 1989; MMWR 1991). Clinicians and scientists often grapple with the best way to understand and communicate risk, as has been demonstrated with basic and well-accessed information such as cancer risks (Woloshin et al. 2002).

Although the scientific community’s ability to communicate statistics and other mathematical concepts needs improvement, a larger issue is the striking lack of numeracy in the general population (Steen 1990). This is the background reality against which findings are communicated. Moreover, even when scientists attempt to communicate statistics accurately, these data can be misconstrued or miscommunicated by popular media and the web, or are in a form that is difficult for the general public to understand and interpret (Schwartz and Woloshin 2004; Woloshin et al. 2003). Methods for communicating scientific information to the public, especially pertaining to human health risk, have been addressed previously, and researchers in the field of biomonitoring should be familiar with this literature (ATSDR 2001).

#### For biomonitoring studies addressing health effects, investigators should distinguish between statistically significant effects and clinically significant effects.

Although the regulator or epidemiologist may be interested in the former, for clinicians and the public in general the latter is of primary interest. For example, studies reporting on the effects of polychlorinated dibenzodioxins, polychlorinated dibenzofurans, and coplanar polychlorinated biphenyls on breastfed infants provided data on levels of these environmental chemicals in the mothers’ milk samples and on infant serum levels of triiodothyronine, thyroxine, thyroid-stimulating hormone, thyroxine-binding globulin, and lymphocyte subsets (Nagayama et al. 1998a, 1998b). Effects on infant serum levels were noted, but no interpretive information was given. While this method of presenting study results is sufficient for many scientists, for the lay audience and others, additional information is desirable. For example, it is not clear whether observable adverse health effects in the infants would be anticipated or whether the serum levels were within clinically normal ranges (LaKind et al. 2004). A small biologic change in a quantitative measure can be statistically significant without necessarily indicating a change that is physiologically or toxicologically significant in terms of adverse health outcomes.

#### The development of an accessible Internet-based site for human biomonitoring data should be a high priority.

This type of site would allow scientists and others to share and compare data obtained from biomonitoring research (Waters and Fostel 2004). Progress with this type of public database is being made in the field of toxicogenomics, which has witnessed the development of data-exchange standards and guidelines for harmonization in data collection (Waters and Fostel 2004). A possible model for such a database is the Chemical Effects in Biological Systems knowledgebase (http://cebs.niehs.nih.gov). Models for standard-setting include the Clinical Data Interchange Standards Consortium (CDISC 2004) and the Standards for Exchange of Nonclinical Data (SEND 2004). The database for human biomonitoring studies should include the research data and reference ranges (both for population concentration data and clinical reference ranges). This type of site will require continual updating, which necessitates a long-term commitment of resources.

#### An established architecture is needed to communicate the meaning of individual and population-based biomonitoring results.

A concerted effort is needed to educate clinicians regarding the availability of expertise in interpreting human biomonitoring data. For most environmental chemicals, it is inappropriate to suggest that individuals consult their doctors with questions about biomonitoring data. Clinicians, especially general practitioners, are often ill prepared to answer specific questions regarding chemical exposures and health risks, nor are there obvious or readily available resources for them to obtain this information at a level and in a form that they and their patients can understand. One current option is to query a poison control center, which would likely refer them to a specialist from an established network of clinical toxicologists. Another available option is to contact an ATSDR office or a Pediatric Environmental Health Specialty Unit (PEHSU).

A nationwide effort is needed to inform physicians of the availability of medical toxicologists, such as members of American College of Medical Toxicology (American College of Medical Toxicology 2005). Efforts have been made to create electronic networks for rural physicians, such as the Rural Physicians Health Network, which links them to larger medical centers and other sources of specialized information that they can use both for specific patient inquiries and for continuing education. Such a network could be established to link physicians more generally to clinical toxicologists and public health officials that would allow them to tap into the growing knowledge base from biomonitoring, epidemiology, and similar studies. This could also provide physicians with appropriate information resources for deciding whether and how to analyze specific environmental chemicals in an individual, how to select the appropriate sample matrix and laboratory, and how to interpret the results.

#### Resources are the limiting factor in creating a robust and continuously updated database of human levels of environmental chemicals, linked to information that would allow these levels to be interpreted in terms of potential health effects.

The panel recommends that resources for this purpose be given a high priority. The panel recognizes that the resources required to build such a database will be significant. However, for protection of public health, it is insufficient to develop a biomonitoring database without the ability to interpret that database. Therefore, the panel recommends that funding agencies and organizations devote the resources necessary for this endeavor.

Because current exposures to certain environmental chemicals may be related to future adverse health effects, the panel recommends that an architecture be developed to support long-term storage of human specimens and that a process be established to provide for sharing of specimens as part of future investigations.

In the shorter term, physicians and others involved in health care require current information on interpretation of human biomonitoring data. Resources such as the American College of Medical Toxicology (2005) and, for pediatric issues, the PEHSUs (ATSDR 2005) are available but are not yet widely recognized. A concerted effort is required to increase the visibility of these resources and to develop additional resources for the effective communication of the interpretation of biomonitoring data.

In addition to resources for health care providers, there is a serious need for well-written, multilingual articles for the lay audience on an array of topics that would assist in improving the public’s ability to understand human biomonitoring information, including associated uncertainties and limitations. In addition to written material (i.e., manuscript-style documents), formats such as pamphlets, posters, graphic narratives, and videos are useful for reaching a wider audience.
